# Assessing the impact of a health navigator on improving access to care and addressing the social needs of palliative care patients experiencing homelessness: A service evaluation

**DOI:** 10.1177/02692163221146812

**Published:** 2022-12-28

**Authors:** Lilian Robinson, Leeann Trevors Babici, Alissa Tedesco, Donna Spaner, Trevor Morey, Naheed Dosani

**Affiliations:** 1 Department of Family and Community Medicine, University of Toronto, Toronto, ON, Canada; 2 Palliative Education and Care for the Homeless Program, Toronto, ON, Canada; 3 Second Mile Club, Kensington Health, Toronto, ON, Canada

**Keywords:** Palliative care, community services, social impact, health impact assessment, outcome measures, homelessness, health equity, social workers

## Abstract

**Background:**

Health navigators are healthcare professionals who specialize in care coordination, case management, navigating transitions, and reducing barriers to care. There is limited literature on the impact of health navigators on community-based palliative care for people experiencing homelessness.

**Aim:**

We devised key performance indicators in nine categories with the aim to quantify the impact of a health navigator on the delivery of palliative care to patients experiencing homelessness.

**Design:**

Data were collected prospectively for all patient encounters involving a health navigator from July 2020 to 2021 and reviewed to determine the distribution of the health navigator’s role and the ways in which patient care was impacted.

**Setting and participants:**

This study was conducted in Toronto, Ontario with the Palliative Education and Care for the Homeless (PEACH) Program. At any one time, the PEACH health navigator served a total of 50 patients.

**Results:**

We identified five key areas of the health navigator role including (1) facilitating access (2) coordinating care (3) addressing social determinants of health (4) advocating for patients, and (5) counselling patients and loved ones. The health navigator role was split evenly between activities pertaining to palliative care for structurally vulnerable populations and community-based palliative care for the general population. To achieve high impact outcomes, a considerable investment of time and energy was required of the health navigator, speaking to the importance of adequate and sustainable funding.

**Conclusions:**

These findings underscore the potential for health navigators to add value to community-based palliative care teams, especially those caring for structurally vulnerable populations.

**What is already known about the topic?**

- Health navigators positively impact the patient experience of palliative care through coordination of care, case management, navigating transitions, and reducing barriers to care.

**What this paper adds?**

- A professional health navigator can improve access to care and help meet the social needs of palliative care patients experiencing homelessness, and the impact of this can be measured.

**Implications for practice, theory or policy**

- Community-based palliative care teams may consider incorporating a professional health navigator into their model to improve access to care, and doing so may lead to wider societal impacts to support structurally vulnerable populations.

## Background

Amongst individuals experiencing homelessness, specific groups are overrepresented, including Indigenous and racialized individuals, people who first experienced homelessness in their youth, people who have experienced foster care, and individuals who identify as 2SLGBTQ+.^1^ Research has clearly shown that people experiencing homelessness are at increased risk for illness and mortality, and there are significant systemic barriers to them receiving good palliative care at the end of life.^2–5^

A health navigator is a healthcare professional or peer who specializes in coordination of care, case management, navigating healthcare transitions, and reducing barriers to care.^6–14^ Multiple studies have demonstrated the positive impact of social workers on palliative care teams, including their capacity to improve quality of care, facilitate discussions about advance care planning, educate patients and families, help patients achieve prognostic alignment, screen for bereavement needs, and lead team discussions.^15–21^ Moreover, there is research demonstrating that health navigators can improve the health care of underserved patients.^22^ However, there is little research to date on the potential for social workers as health navigators to facilitate equitable access to palliative care amongst structurally vulnerable populations, and to our knowledge there are no studies demonstrating the impact of a professional health navigator in community-based palliative care for individuals experiencing homelessness.

The Palliative Education and Care for the Homeless (PEACH) program is a community-based palliative care program in Toronto, ON developed in response to a need for the delivery of equity-oriented palliative care to individuals experiencing homelessness.^23^ At the beginning of the COVID-19 pandemic, PEACH received funding to establish a health navigator role with the aim of providing social care through addressing the housing, income, food security and access to health care needs of this patient population. An individual with a Master of Social Work was hired for this role given their specialized skills in systems navigation, interdisciplinary collaboration, and evaluating and meeting the social needs of their clients.

In this study, we sought to characterize the activities of health navigator with a Master of Social Work delivering palliative care to individuals experiencing homelessness, and evaluate the impact of a health navigator on the delivery of community-based palliative care to people experiencing homelessness in Toronto, ON. We hypothesized that over a 1-year period the addition of a health navigator to the PEACH program would facilitate access to palliative care and social services as well as help overcome structural barriers to health and well-being for people experiencing homelessness while receiving palliative care.

## Methods

### Design of service evaluation

For the purposes of this study, an original service evaluation tool was designed collaboratively by the authors to capture the roles and responsibilities of the PEACH health navigator. Unique key performance indicators (KPIs) were devised for the purposes of evaluation and categorized into one of two domains, including KPIs applicable to (1) palliative care for people experiencing homelessness and (2) general community- and home-based palliative care, or “mainstream” palliative care. KPIs in Domain One were derived from the PEACH health navigator job description, while those in Domain Two were derived from current literature pertaining to the role of a health navigator in general palliative care settings, including both community- and home-based settings.^15–21,24^ A total of nine KPI categories and 51 individual KPIs were included (Table 1).

**Table 1.** Key performance indicators (KPIs) to measure the impact of a health navigator in community-based palliative care for people experiencing homelessness.

Key performance indicators by domain and category			
Domain One: People experiencing homelessness or living in vulnerable housing	Category One: Housing	Domain Two: General community-based palliative care	Category Five: Accompaniment to medical appointments
	KPI 1.1 Referral to shelter-hotel program		KPI 5.1 Attended health appointment with client
	KPI 1.2 Housing application discussion		KPI 5.2 Accompanied to PCU admission
	KPI 1.3 Housing application submitted		KPI 5.3 Facilitated virtual care appointment
	KPI 1.4 Housing application advocacy		Category Six: Access to interdisciplinary care
	KPI 1.5 Accompany to view housing offer		KPI 6.1 Connected to dentist or optometrist
	KPI 1.6 Housing secured		KPI 6.2 Connected to occupational or physiotherapy
	KPI 1.7 Housing maintained >12 months		KPI 6.3 Connected to addictions support worker or case manager
	KPI 1.8 Eviction prevented		KPI 6.4 Aided in referral to specialist
	KPI 1.9 Advocacy to maintain housing		KPI 6.5 Coordinated with specialist
	KPI 1.10 Arrange extreme clean or pest control treatment		Category Seven: Counselling and psychosocial support
	KPI 1.11 Arrange maintenance visit or place work order request		KPI 7.1 Counselling provided
	Category Two: Income		KPI 7.2 Referral made to mental health provider
	KPI 2.1 Application for ODSP/CPP-D		Category Eight: Capacity building or partnership development
	KPI 2.2 Secured ODSP/CPP-D		KPI 8.1 Conducted presentation about PEACH
	KPI 2.3 Application for ODSP Transportation Coverage		KPI 8.2 Involved in media respresentation of PEACH
	KPI 2.4 Application for ODSP Medical Necessities Benefit		KPI 8.3 Quantifiably extended reach
	KPI 2.5 Application for shelter allowance		KPI 8.4 Facilitated grief circle for community partners
	KPI 2.6 Application for Housing Stabilization Fund		Category Nine: Patient advocacy and health outcomes
	KPI 2.7 Assist with client finances		KPI 9.1 Optimized care by attending case conference meeting
	Category Three: Food (In)Security		KPI 9.2 Prevented adverse health event through surveillance
	KPI 3.1 Connected to Food Bank		KPI 9.3 Affected positive change in care through surveillance
	KPI 3.2 Connected to Meals on Wheels		KPI 9.4 Facilitated symptom management conversation
	KPI 3.3 Connected to grocery gift card program		KPI 9.5 Documented advance care planning discussion
	KPI 3.4 Advocacy regarding food security at case conference		
	Category Four: Referrals and coordination		
	KPI 4.1 Hospice Toronto visitor volunteer referral		
	KPI 4.2 Referral made to Good Wishes Project		
	KPI 4.3 Wheel Trans application		
	KPI 4.4 Connected client with PAID ID clinic		
	KPI 4.5 Facilitated health card renewal		
	KPI 4.6 Coordinating safety planning		
	KPI 4.7 Coordinating medication pick up and delivery		
	KPI 4.8 Led discussion with family		
	KPI 4.9 Coordinated taxi or wheel trans booking		
	KPI 4.10 Coordinated other (i.e. LHIN services)		

PCU: palliative care unit; ODSP: Ontario disability service pension; CPP-D: Canadian pension plan – Disability benefit.

### Setting and population

This study was conducted in Toronto, ON, with study approval granted by the Inner City Health Associates research oversight committee. The PEACH health navigator position was occupied by one individual throughout the duration of the study. At any one time, the health navigator served a total of 50 patients experiencing homelessness. While there are many definitions of homelessness, PEACH considers an individual to be experiencing homelessness if they are sleeping outside, couch-surfing, living in shelter, or vulnerably housed in temporary or permanent housing.

### Data collection

KPIs were tracked prospectively by the PEACH health navigator between July 2020 and July 2021. Each time the health navigator carried out an activity or achieved an outcome corresponding to a KPI, it was logged in an anonymized excel spreadsheet for tabulation. These encounters were also documented in the patients’ electronic medical record.

### Data analysis

All data points were tabulated to calculate the total number of times each individual KPI was achieved as well as the total number of occurrences logged per KPI category. KPIs were further categorized by rate of completion, including those completed at low frequency (0–1 times), moderate frequency (2–9 times) and high frequency (10+ times) during the study period.

Collated data were then interpreted to understand the distribution of the health navigator’s role and examine its impact on the delivery of palliative care to individuals experiencing homelessness. The health navigator role was further characterized using three distinct methods of analysis, which are detailed in the supplementary material (Supplemental File 1). KPIs are considered a direct reflection of the health navigator’s activities, and are therefore referred to as “activities” in both the results and discussion sections.

## Findings

Between July 2020 and July 2021, the PEACH health navigator logged a total of 407 activities reflecting the KPIs devised to capture their role. In the domain of providing palliative care to people experiencing homelessness, the health navigator completed a total of 207 activities, and in the category of general community-based palliative care, or “mainstream” palliative care, a total of 200. The health navigator’s role encompassed all overarching categories to varying degrees, including referrals and coordination (77), accompaniment to medical appointments (72), housing (60), patient advocacy and health outcomes (59), income (51), access to interdisciplinary care (35), counselling and psychosocial support (25), food (in)security (19), and capacity building and partnership development (9) (Table 2).

**Table 2.** Key performance indicator (KPI) by total number of events per category and KPIs per frequency category.

KPI Category		Total Number of Events	Number of KPIs per Frequency Category		
			High^a^	Moderate^b^	Low^c^
Domain One: People experiencing homelessness	Housing	60	1	8	2
	Income	51	2	6	1
	Food (In)security	19	1	0	3
	Referrals and Coordination	77	2	6	2
	Total	207			
Domain Two: Community-based palliative care	Accompaniment to Medical Appointment	72	1	1	1
	Access to Interdisciplinary Care	35	1	2	2
	Counselling and Psychosocial Support	25	1	0	1
	Capacity Building and Partnership Development	9	0	2	2
	Patient Advocacy and Health Outcomes	59	1	2	2
	Total	200			

a High frequency: 10 + events.

b Moderate frequency: 2–9 events.

c Low frequency: 0–1 events.

Specific activities completed most often by the health navigator included booking a taxi or medical transportation (10), applying for transportation coverage (11), applying for a medical necessities benefit through provincial social services programs (12), advocating for a patient in their application for housing (14), connecting individuals to a local hospice grocery program (17), coordinating with specialists (23), providing counselling to patients and their loved ones (25), picking up and delivering medication (32), attending case conference meetings (52), and attending an appointment with a patient (69) (Table 2).

## Discussion

This study adds to a growing body of literature characterizing the role of a health navigator in community-based palliative care and fills a gap in the area of impact measurement. We highlight the ways in which a health navigator with a background in social work can take a focused approach to the social determinants of health to facilitate access to equitable care at the end of life.

Emerging research offers similar emphasis on addressing the social determinants of health to improve access to palliative care for people living with life limiting illness and experiencing structural vulnerability,^3–5,23^ including through harm-reduction strategies^25–29^ and better integration of social services with health care services.^21,30–32^ We argue that addressing the social needs of patients and providing a person-centered approach to care is of considerable value not only in the field of socially-oriented palliative care for structurally vulnerable populations but in all fields of healthcare delivery. In fact, it is now widely recognized that addressing the social determinants of health is vital for improving overall health and reducing health disparities.^33,34^ For community-based palliative care teams that identify a gap in meeting the social needs of their patient population and wish to establish a health navigator position, we also provide an objective framework for evaluating the impact of a professional health navigator in community-based palliative care settings with the use of key performance indicators (KPIs).

Previous studies examining the impact of social workers and health navigators on the delivery of palliative care have focused primarily on outcomes such as pain management, advanced care planning documentation, and hospice utilization.^15,17,18^ In contrast to the existing literature, our study is unique in its measurement of outcomes pertaining to the social determinants of health. By examining the rates at which these outcomes were completed during the study period, we found that a high degree of output was required from the health navigator to achieve meaningful outcomes related to social needs (Supplemental File 1). For instance, the completion of 28 activities related to housing (i.e. housing applications and related advocacy) led to two successful housing applications during the study period, which although representative of a small number of outcomes, presumably led to considerable impact on the health and wellbeing of those patients who were housed in the context of a life-limiting illness. Given the well-documented challenges of overcoming structural barriers to facilitating dignifying and equitable end-of-life care, we encourage teams to acquire adequate and sustainable funding for a professional health navigator to maximize their potential impact.^5,21,31^

This study was limited by the fact that we had no baseline data or control group for comparison. Therefore, although we can postulate that a professional health navigator had a positive impact on access to palliative care delivered to this population, we cannot comment on the degree to which various outcomes related to the social determinants of health would have been achieved in the absence of a health navigator. Likewise, these findings may not be broadly applicable to other communities within a different health network if the specific needs of the population in question differ considerably from the population we studied. Certain outcomes were achieved at a lower rate than anticipated, which may speak to the difficulty of measuring these outcomes, highlight an area for role development, or it may indicate that a longer study period is needed to capture outcomes that were achieved at low frequency over the course of 1 year of data collection.

This study opens the door for future avenues of research to enrich our understanding of the potential for a health navigator to enhance the delivery of palliative care to structurally vulnerable populations as well as those accessing “mainstream” community-based palliative care services. Future directions may include measuring the impact of a health navigator on quality of care, patient outcomes, survival, and cost savings for the healthcare system as a result of addressing the social determinants of health.

## Supplemental Material

sj-pdf-1-pmj-10.1177_02692163221146812 – Supplemental material for Assessing the impact of a health navigator on improving access to care and addressing the social needs of palliative care patients experiencing homelessness: A service evaluation Click here for additional data file. Supplemental material, sj-pdf-1-pmj-10.1177_02692163221146812 for Assessing the impact of a health navigator on improving access to care and addressing the social needs of palliative care patients experiencing homelessness: A service evaluation by Lilian Robinson, Leeann Trevors Babici, Alissa Tedesco, Donna Spaner, Trevor Morey and Naheed Dosani in Palliative Medicine
