# Supplementary material for: Assessing the impact of a health navigator on improving access to care and addressing the social needs of palliative care patients experiencing homelessness: A service evaluation
Source: Palliat Med. 2022 Dec 28;37(4):646–51. doi: 10.1177/02692163221146812 (PMC10074742; doi:10.1177/02692163221146812)
Supplement: sj-pdf-1-pmj-10.1177_02692163221146812 – Supplemental material for Assessing the impact of a health navigator on improving access to care and addressing the social needs of palliative care patients experiencing homelessness: A service evaluation [file sj-pdf-1-pmj-10.1177_02692163221146812.pdf]

<u>Supplementary File One</u>

Measuring the Impact of a Health Navigator on a Community-Based Palliative Care Team

Section 1.1: Description of Supplementary Materials 1

Section 1.2: Methods - Impact Measurement 1

Section 1.2.1 Impact Measurement Method One: Rate of KPI Occurrence within KPI Categories 2

Table Two: Key Performance Indicator (KPI) by total number of occurrences per category and KPIs per frequency category 3

Section 1.2.2 Impact Measurement Method Two: KPI Occurrence by Category and Measurement Type 4

Supplementary Table One: Key Performance Indicators (KPI) by domain and measurement type 4

Section 1 2.3 Impact Measurement Method Three: Rate of KPI Occurrence by Frequency Category and Measurement Type 5

Supplementary Table Two: Key Performance Indicator (KPI) by frequency category and measurement type 5

Section 1.1: Description of Supplementary Materials

As specified in the body of the main article, we devised key performance indicators to capture the roles and responsibilities of the PEACH health navigator, and categorized into one of two domains, including KPIs applicable to (1) palliative care for people experiencing homelessness and (2) general community- and home-based palliative care, or 'mainstream' palliative care. In addition to the interpretations of the data included in the main article, we further characterized the health navigator role by analyzing collated data using three distinct methods of analysis, which are detailed here in the supplementary material. We include these methods of analysis for community-based palliative care teams that identify a gap in meeting the social needs of their patient population and wish to establish a health navigator position. We hope this will serve as an objective framework for evaluating the impact of a professional health navigator in community-based palliative care settings with the use of key performance indicators (KPIs).

Section 1.2: Methods - Impact Measurement

Impact was measured using three distinct methods of analysis, which were derived through careful and repeated interpretation of the data by the principal author. Method one involved tabulating the total number of KPIs completed at low, moderate, and high frequency within each KPI category. Method two involved tabulating the total number of KPIs completed at

low, moderate, or high frequency, then further categorizing KPIs by their measurement type (i.e. working vs. outcome). Method three involved totalling the number of occurrences per KPI category, then categorizing by measurement type (i.e. working vs. outcome).

In addition to quantifying the rate of KPI completion across and within domains of the health navigator role, we also determined how often KPIs were completed based on their designation as ‘working’ or ‘outcome’ measures. This was done to better understand the measurable impact of the health navigator role on the delivery of palliative care to patients experiencing structural vulnerabilities. Working measures were defined as activities performed in service of a potential outcome, however it was understood in our analysis that the working measure itself may impact patient care directly even if a corresponding downstream outcome was either not measured or not realised during the study period. Framing the KPIs in this way was helpful to understand the amount of work required to bring about an outcome, particularly those related to the social determinants of health, which we anticipated would require a greater investment of time

Each KPI was designated as an outcome or working measure, with a working measure being defined as an action in service of an outcome, and an outcome measure being defined as an achievement that occurred as a result of a working measure. For example, the KPI ‘applied for provincial health funding’ is categorized as a working measure, while ‘secured provincial health funding’ is an outcome measure. Notably, although working measures are necessary for procuring a greater outcome, working measures themselves may represent an impact brought about by the health navigator.

Section 1.2.1 Impact Measurement Method One: Rate of KPI Occurrence within KPI Categories

Individual KPIs were completed at varying rates across all categories. A total of 16 individual KPIs were completed at low frequency (0-1 times), 27 KPIs at moderate frequency (2-9 times), and 10 KPIs at high frequency (10+ times) (Table 2).

Individual KPIs with occurrences of ten or more included booked taxi or medical transportation (10), applied for transportation coverage (11), applied for medical necessities benefit through provincial social services program (12), advocated for housing application (14), connected to local Hospice grocery program (17), coordinated with specialists (23), provided counselling (25), picked up and delivered medication (32), attended case conference meeting (52), and attended an appointment with a patient (69) (Table 2).

Individual KPIs achieved least often, only 0-9 times, came from all KPI categories, meaning there were activities within each category of the health navigator's roles and responsibilities that were completed infrequently. Individual KPIs with the fewest number of occurrences included referral to a shelter hotel program (1), arranged pest control treatment (1), secured income support through Provincial support program (0), connected to home meal delivery program (1), connected to a food bank (0), referred to a Hospice volunteer (1), submitted Wheel Trans application (0), accompanied client to Palliative Care Unit (PCU) admission (1), connected to addictions worker or intensive case manager (1), and aided in referral to specialist (0). (Table 2)

KPIs achieved at moderate (2-9 times) and high (10+ times) degrees of frequency were broadly represented across all categories. Moderate frequency KPIs were mostly concentrated in the areas of housing, referrals and care coordination, and income supports. High frequency KPIs were established in 8 of 9 categories, excluding capacity building and partnership development (Table 2).

Table Two: Key Performance Indicator (KPI) by total number of occurrences per category and KPIs per frequency category

KPI Category		Total Number of Events	Number of KPIs per Frequency Category		
			High[a]	Moderate[b]	Low[c]
Domain One: People experiencing homelessness	Housing	60	1	8	2
	Income	51	2	6	1
	Food (In)security	19	1	0	3
	Referrals and Coordination	77	2	6	2
	**Total**	**207**			
Domain Two: Community-based palliative care	Accompaniment to Medical Appointment	72	1	1	1
	Access to Interdisciplinary Care	35	1	2	2
	Counselling and Psychosocial Support	25	1	0	1
	Capacity Building and Partnership Development	9	0	2	2
	Patient Advocacy and Health Outcomes	59	1	2	2
	**Total**	**200**			

[a]High Frequency: 10+ events [b]Moderate Frequency: 2-9 events [c]Low Frequency: 0-1 events

Section 1.2.2 Impact Measurement Method Two: KPI Occurrence by Category and Measurement Type

In the domain of KPIs pertaining to palliative care for people experiencing homelessness, a total of 207 occurrences were achieved, of which 99 (48%) were categorized as working type KPIs, and 108 (52%) were categorized as outcome type. Within this domain, income was the KPI category with the highest ratio of working:outcome type KPI occurrences (51:0). KPIs pertaining to housing were also largely working type (72%) rather than outcome (28%). In contrast, occurrences in the areas of food (in)security and referrals and coordination were predominantly outcomes, with 95% of occurrences corresponding to an outcome in both categories.

In the domain of general community-based palliative care, a total of 200 occurrences were achieved, of which a greater proportion were working KPIs (88%) than outcome (12%). Of the categories in this domain, two were marked by 100% working KPIs, including accompaniment to medical appointments and counselling and psychosocial support, with no measurable outcomes attached. Capacity building and partnership development was the only category in the domain of general community-based palliative care that featured a higher percentage of outcome KPI occurrences than working KPIs (Supplementary Table 1).

Supplementary Table One: Key Performance Indicators (KPI) by domain and measurement type

KPI Category		Total Number of Events	Number of KPIs by Measurement Type (% of total)	
			Working	Outcome
Domain One: People experiencing homelessness	Housing	60	43 (72%)	17 (28%)
	Income	51	51 (100%)	0 (0%)
	Food (In)security	19	1 (5%)	18 (95%)
	Referrals and Coordination	77	4 (5%)	73 (95%)
	**Total**	**207**	**99 (48%)**	**108 (52%)**
Domain Two: Community-based palliative care	Accompaniment to Medical Appointment	72	72 (100%)	0 (0%)
	Access to Interdisciplinary Care	35	23 (66%)	12 (34%)
	Counselling and Psychosocial Support	25	25 (100%)	0 (0%)
	Capacity Building and Partnership Development	9	3 (33%)	6 (67%)
	Patient Advocacy and Health Outcomes	59	52 (88%)	7 (12%)
	**Total**	**200**	**175 (88%)**	**25 (12%)**

Section 1 2.3 Impact Measurement Method Three: Rate of KPI Occurrence by Frequency Category and Measurement Type

Recalling that working KPIs represent an action in service of an outcome, and outcome KPIs an achievement that occurred as a result of work completed, we found that KPIs completed at low frequency (0-1 times), were predominantly outcome type KPIs (75%). Contrastingly, KPIs completed at high frequency (10+ times), were largely working type KPIs (70%). KPIs achieved at moderate frequency (2-9 times) were equally likely to be working type (48%) as they were outcome (52%). If pooled together, low and moderate frequency KPIs - those which occurred 0-9 times - were 60% outcome and 40% working type (Supplementary Table Two).

Supplementary Table Two: Key Performance Indicator (KPI) by frequency category and measurement type

KPI Frequency Category	Total number of KPIs	Number of KPIs by Measurement Type (% of total)	
		Working	Outcome
Low (0-1 events)	16	4 (25%)	12 (75%)
Moderate (2-9 events)	27	13 (48%)	14 (52%)
High (10+ events)	10	7 (70%)	3 (30%)
